# Clinical characteristics of patients with central nervous system relapse in BCR-ABL1-positive acute lymphoblastic leukemia: the importance of characterizing ABL1 mutations in cerebrospinal fluid

**DOI:** 10.1007/s00277-017-3002-1

**Published:** 2017-04-27

**Authors:** Ricardo Sanchez, Rosa Ayala, Rafael Alberto Alonso, María Pilar Martínez, Jordi Ribera, Olga García, José Sanchez-Pina, Santiago Mercadal, Pau Montesinos, Rodrigo Martino, Pere Barba, José González-Campos, Manuel Barrios, Esperanza Lavilla, Cristina Gil, Teresa Bernal, Lourdes Escoda, Eugenia Abella, Ma Luz Amigo, Ma José Moreno, Pilar Bravo, Ramón Guàrdia, Jesús-María Hernández-Rivas, Antoni García-Guiñón, Sonia Piernas, José-María Ribera, Joaquín Martínez-López

**Affiliations:** 1 Instituto de Investigación Hospital 12 de Octubre (i+12), Servicio de Hematología, Hematología Traslacional, Hospital Universitario 12 de Octubre, Avda de Andalucía s/n, 28041 Madrid, Spain; 2 Institut de Recerca contra la Leucèmia Josep Carreras, ICO-Hospital Germans Trias i Pujol, Badalona, Spain; 3 ICO-Hospital Duran i Reynals (Bellvitge), Barcelona, Spain; 4 Hospital Universitari i Politècnic La Fe, Valencia, Spain; 5 Hospital de la Santa Creu i Sant Pau, Barcelona, Spain; 6 Hospital Universitari Vall d’Hebron, Barcelona, Spain; 7 Hospital Universitario Virgen del Rocío, Sevilla, Spain; 8 Hospital Regional Universitario Carlos Haya, Málaga, Spain; 9 Hospital Universitario Lucus Augusti, Lugo, Spain; 10 Hospital General Universitario de Alicante, Alicante, Spain; 11 Hospital Universitario Central de Asturias, Oviedo, Spain; 12 Hospital Universitari Joan XXIII, Tarragona, Spain; 13 Hospital del Mar, Barcelona, Spain; 14 Hospital General Universitario Morales Meseguer, Murcia, Spain; 15 Hospital Universitario Virgen de la Victoria, Málaga, Spain; 16 Hospital de Fuenlabrada, Fuenlabrada, Madrid, Spain; 17 ICO-Hospital Universitari Dr. Josep Trueta, Girona, Spain; 18 Hospital Universitario de Salamanca, Salamanca, Spain; 19 Hospital Universitari Arnau de Vilanova, Lleida, Spain; 20 Hospital Universitari Parc Taulí, Sabadell, Barcelona, Spain

**Keywords:** Neoplasia, Acute lymphoblastic leukemia relapse, Central nervous system, Mutation analysis, BCR-ABL1

## Abstract

We investigated the frequency, predictors, and evolution of acute lymphoblastic leukemia (ALL) in patients with CNS relapse and introduced a novel method for studying BCR-ABL1 protein variants in cDNA from bone marrow (BM) and cerebrospinal fluid (CSF) blast cells. A total of 128 patients were analyzed in two PETHEMA clinical trials. All achieved complete remission after imatinib treatment. Of these, 30 (23%) experienced a relapse after achieving complete remission, and 13 (10%) had an isolated CNS relapse or combined CNS and BM relapses. We compared the characteristics of patients with and without CNS relapse and further analyzed CSF and BM samples from two of the 13 patients with CNS relapse. In both patients, classical sequencing analysis of the kinase domain of BCR-ABL1 from the cDNA of CSF blasts revealed the pathogenic variant p.L387M. We also performed ultra-deep next-generation sequencing (NGS) in three samples from one of the relapsed patients. We did not find the mutation in the BM sample, but we did find it in CSF blasts with 45% of reads at the time of relapse. These data demonstrate the feasibility of detecting BCR-ABL1 mutations in CSF blasts by NGS and highlight the importance of monitoring clonal evolution over time.

## Introduction

About 25% of adults and about 5% of children with acute lymphoblastic leukemia (ALL) have the Philadelphia chromosome or the BCR-ABL1 rearrangement. The BCR-ABL1 genetic fusion typically encodes a 190-kDa constitutively active tyrosine kinase protein, but the protein is 210 kDa in some cases. Although the prognosis of ALL has improved with the introduction of tyrosine kinase inhibitor (TKI) therapy, relapses still occur, especially in elderly patients.

The CNS is frequently a reservoir for tumor cells that avoid exposure to therapeutic agents. The frequency of CNS relapse in BCR-ABL1-positive ALL ranges from 8 to 17% [1], and the overall survival (OS) of these patients is shorter than that of patients without CNS involvement [2]. Since the introduction of TKIs [3], intensive chemotherapy plus therapeutic treatment with a TKI has become the first-line treatment in such patients [4, 5], with imatinib being the most extensively used TKI. However, the imatinib concentration in CNS is low, which may explain, at least in part, the frequency of CNS relapse despite adequate CNS prophylaxis [6]. Accurate monitoring of disease status during TKI therapy is mandatory, and it is appropriate to adjust the dose and the type of TKI in cases in which there is poor tolerability or the appearance of mutations.

Until very recently, Sanger sequencing (SS) was the most widely used method for evaluating the kinase domain mutation status of the chimeric BCR-ABL1 protein. However, the development of next-generation sequencing (NGS) technologies has overcome some of the limitations of SS, allowing sequencing of the entire kinase domain thousands or even millions of times at once. Ultra-deep NGS allows several sequence reads, each one corresponding to a single DNA clone, which detect the sequence at a single nucleotide position multiple times with high sensitivity [7].

Our primary goal was to study the frequency, predictors, and evolution of the disease in patients with CNS relapse in two consecutive clinical trials of Philadelphia chromosome-positive ALL that were conducted by the Spanish PETHEMA (Programa Español de Tratamientos en Hematología) Group. A secondary aim was to evaluate the use of a novel method for studying the pathogenic mechanisms and variants of uncertain significance (VUS) in the BCR-ABL1 kinase domain using cDNA from samples of bone marrow (BM) and CSF blasts with the idea of selecting optimal TKI treatment based on clonal evolution from BM to CSF cells.

## Materials and methods

### Patients

We reviewed the CNS relapse data of patients who were included in two consecutive clinical trials, LAL-OPH-2007 [8] and LAL-PH-2008 [9], which were performed by the PETHEMA Group for patients with Philadelphia chromosome-positive ALL who were older or younger than 55 years, respectively. All patients received the TKI imatinib in combination with chemotherapy, and CNS prophylaxis consisted of triple intrathecal therapy (TIT) with methotrexate cytarabine and hydrocortisone. These studies were conducted in accordance with the principles of the Declaration of Helsinki, and the protocols were approved by the appropriate institutional review boards. All patients provided written informed consent for the analysis of their specimens.

We evaluated BM and CSF samples from two patients that were taken either at diagnosis or at recurrence. Patient 1 was treated within the LAL-PH-2008 protocol, and patient 2 was treated according to the LAL-OPH-2007 protocol.

### Determination of BCR-ABL1 kinase mutations in CSF

RNA was extracted from fresh BM using a standard TRIzol® reagent-based protocol. For the CSF specimen, RNA was extracted using the RNeasy® Mini Kit (Qiagen, Hilden, Germany). cDNA was obtained using the High-capacity cDNA Reverse Transcription Kit (Thermo Fisher, Palo Alto, CA). The kinase domain of the cDNA was amplified in two steps from the following samples: BM at diagnosis and BM or CSF at relapse. In the first step of amplification, we used 5′-CTGGCCCAACGATGGCGA-3′ (for BCR exon 1) and 5′-GTACTCACAGCCCCACGGA-3′ (for ABL1 exon 2) as the forward and reverse primers, respectively. This excluded the untranslocated ABL1 allele from the analysis. The kinase domain was amplified from the first PCR product using the primers 5′-AAGCGCAACAAGCCCACTGTCTAT-3′ and 5′-CTTCGTCTGAGATACTGGATTCCTG-3′, which cover the entire kinase domain, from residue Gly^227^ to residue Gly^514^. Both PCR reactions were performed using Taq1b FastStart DNA Master^PLUS^ HybProbe (Roche Applied Science, Mannheim, Germany). Following the manufacturer’s protocols, the amplicons were purified with Agencourt AMPure XP Beads (Beckman Coulter, Inc., Brea, CA) at an initial ratio of 1.8× and visualized using the Bioanalyzer 2100 (Agilent Technologies, Santa Clara, CA). Further enzymatic fragmentation of the kinase domain was performed using Ion ShearPlus (Ion Torrent, Thermo Fisher, Palo Alto, CA) to obtain ∼270-bp fragments. After another identical purification step with AMPure XP Beads, we ligated the adaptors and performed nick repair. Each library was barcoded with Ion Xpress Barcode Adapters (Thermo Fisher). The resulting new fragments were purified with AMPure XP beads (1.2× ratio) and selected with E-Gel® SizeSelect™ agarose gels (Invitrogen™ Thermo Fisher). Later, we re-amplified the fragments, purified them with beads (1.5× ratio), and quantified them with the Ion Library Quantitation Kit (Thermo Fisher). The final barcoded libraries were pooled and adjusted to a final concentration of 50 pM.

The kinase domain libraries theoretically covered almost a third of an Ion Proton Chip, which would provide ∼20 million reads. Template preparation, enrichment, and chip loading were performed using the Ion Chef™ system (Thermo Fisher). Sequencing was carried out on the Ion Proton™ System for next-generation sequencing (Thermo Fisher). The final read data set was analyzed using Torrent Suite Software V.5.0.3 (Thermo Fisher), resulting in two to four million reads per sample. The detected variants were reviewed manually using the Integrative Genomics Viewer (IGV V.2.3.40, Broad Institute, Cambridge, MA).

### Statistical analysis

Baseline characteristics were reported as frequency and percentage for categorical variables and as median and range for quantitative variables. Comparisons of proportions and the medians of variables between different groups were performed using the χ^2^ test, Fisher’s exact test, or the non-parametric median test as appropriate.

OS was measured from the time of diagnosis to the time of the last follow-up or death from any cause. The complete response (CR) duration was defined as the time from the first CR achievement to the relapse date, which was considered an event, or as the last follow-up. Actuarial survival curves were performed using the Kaplan-Meier estimation, and the log-rank test was used for comparisons between groups. Binary logistic regression was used for univariate analysis to study the association of predictive factors with CNS relapse.

Two-sided *P* values <0.05 were considered statistically significant. The statistical package SPSS version 23.0 (Statistical Package for Social Sciences Inc., Chicago, IL) was used for all analyses.

## Results

### Clinical characteristics of patients with CNS relapse

**Fig. 1:**
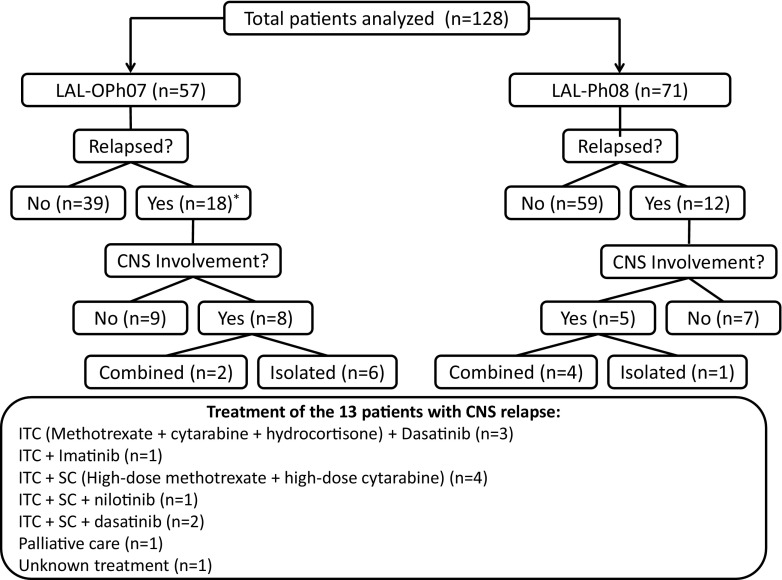
Study flow chart. Treatment details for the ALL patients with central nervous system (*CNS*) relapse. *ITC* intrathecal chemotherapy, *SC* systemic chemotherapy. *One of the relapsed patients had an unknown recurrence

A total of 128 BCR-ABL1-positive ALL patients were analyzed, 57 from the LAL-OPH2007 trial and 71 from the LAL-PH08 trial. Of the 57 LAL-OPH07 patients, 57 (100%) achieved complete remission (CR), 39 (68%) of who had not relapsed at the time of the analysis. All patients from the LAL-PH08 trial also achieved CR, and 59 (83%) had not relapsed at the time of the analysis. Of the LAL-OPH07 patients, 18 (32%) relapsed: 8 had CNS recurrence (isolated in 6), and 10 relapsed either in the BM (*n* = 9) or at an unknown site (*n* = 1). In the LAL-PH08 trial, five out of 12 relapsed patients had combined (*n* = 4) or isolated (*n* = 1) CNS involvement (Fig. 1). Overall, the frequencies of CNS relapse were 7% in the LAL-PH08 trial and 14% in the LAL-OPH07 trial, and a total of 13 patients in the two trials showed CNS involvement at relapse. The main clinical and biological characteristics of the patients in the two clinical trials are summarized in Table 1.

**Table 1:** Summary of the clinical characteristics of the patients included in the PETHEMA LAL-OPH-2007 and the PETHEMA LAL-PH-2008 clinical trials

Protocol/variable	LAL PH08 protocol			LAL OPH07 protocol		
	CNS relapse (*n* = 5)	Relapse outside the CNS (*n* = 7)	No relapse (*n* = 59)	CNS relapse (*n* = 8)	Relapse outside the CNS (*n* = 9)	No relapse (*n* = 39)
Age in years, median (range)	47 (40–53)	39 (23–53)	37 (17–55)	64 (58–82)	65 (57–79)	68 (56–82)
Sex, male/female	3/2	3/4	32/27	3/5	4/5	15/24
WBC count at diagnosis (×10^9^/L), median (range)	16.2 (7.4–211.7)	20.2 (6.1–354.5)	10.6 (1–390)	28.3 (4.5–142)	11 (2.4–139)	7.6 (1.1–409)
MRD level (%) after induction, median (range)	0.001 (0–1.98)	0.2 (0–1.65)	0.01 (0–12)	0.96 (0–2.1)	2.5 (0–3.8)	0.01 (0–17)
Transplantation, Y/N	4/1	6/1	52/0^a^	0/8	0/9	4/35
Bone marrow infiltration at relapse, Y/N	4/1	7/0	–	2/6	9/0	–
Response after relapse	CR: 4 Progression: 1	CR: 3 Progression: 1 Early death: 2 Not evaluated^b^: 1	–	CR: 3 PR: 0 Progression: 2 Early death: 1 Not evaluated^d^: 2	CR: 3 PR: 1 Progression: 1 Early death: 3 Not evaluated^b^: 1	–
Second relapse, Y/N	4/0	2/1^c^	–	1/2	2/1	–
Follow-up time from diagnosis in months, median (range)	39.2 (21, 58.6)	18.4 (12.6, 28.5)	18.7 (1.4, 77.9)	32.6 (11.3, 47.5)	21.9 (8.9, 49.9)	11.2 (1.3, 82.9)
Follow-up time from CNS relapse in months, median (range)	13.1 (8.1, 66.6)	–	–	4.4 (0.6, 22.6)	–	–
Follow-up time between relapses in months, median (range)	7.8 (3–25.3)	7.55 (7–8.1)	–	3.37	4.92 (7.17–2.67)	–

^a^The remaining seven patients were in the consolidation phase of chemotherapy when the data were collected

^b^Palliative treatment

^c^Transplant-related mortality

^d^Stopped due to toxicity: 1; lost to follow-up: 1

*CR* complete remission, *MRD* minimal residual disease, *PR* partial remission, *WBC* white blood cell

**Fig. 2:**
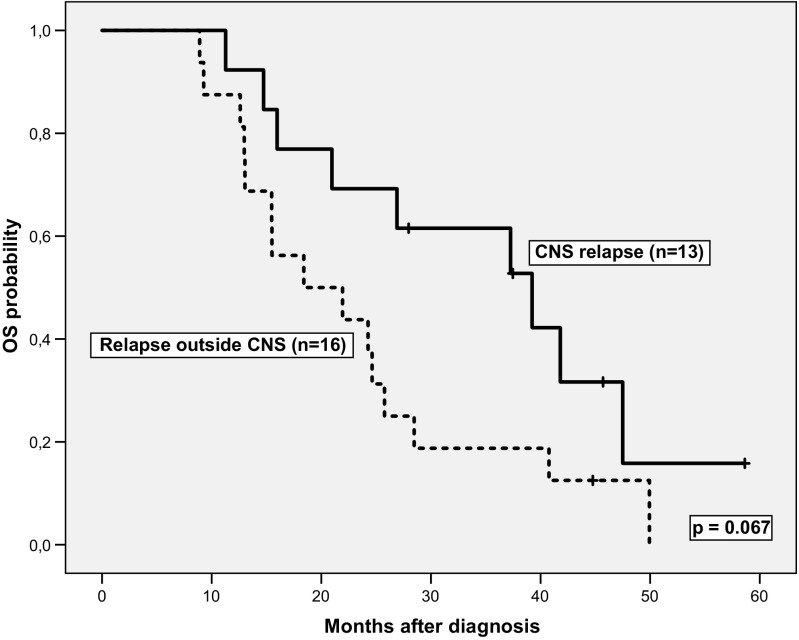
Overall survival curves for the BCR-ABL1 ALL patients with central nervous system (*CNS*) relapse vs. relapse at other sites

Allogeneic hematopoietic stem cell transplantation (HSCT) was performed in 62/71 (87%) patients in the LAL-PH08 trial (two had relapsed before HSCT and the other seven patients were in the consolidation phase of chemotherapy at the time of data review). HSCT was performed in only four (7%) patients in the LAL-OPH07 trial. No statistically significant differences in CR duration were found for cases with CNS relapse and combined or isolated relapse (*n* = 13) vs. patients with relapses at sites other than the CNS (*n* = 16) (median CR duration (95% CI) 22.6 (7.8–37.3) vs. 10.6 (6.5–14.6) months, respectively, *P* = 0.639). The treatment administered to the 13 patients with CNS relapse was as follows: intrathecal chemotherapy (ITC) plus dasatinib (*n* = 3) or imatinib (*n* = 1); ITC and systemic chemotherapy, including high-dose methotrexate (HD-MTX) and high-dose ARA-C (HD-ARAC) (*n* = 4); ITC, HD-MTX, HD-ARAC, and nilotinib (*n* = 1) or dasatinib (*n* = 2); palliative care (*n* = 1); and unknown treatment (*n* = 1). A second allogeneic HSCT was performed in one patient. Of the 13 patients, seven achieved CR, one had an early death, one withdrew from treatment because of toxicity, one was lost to follow-up, and the other three died due to progression. There was a trend for longer OS in patients with CNS relapse vs. those with relapse outside the CNS: median 39.2 (20.8–57.6) vs. 18.4 (5.8–31.0) months, *P* = 0.067 (Fig. 2). Age, sex, WBC count, and MRD status at the end of induction were not associated with the risk of CNS relapse.

### Molecular analysis of CSF and BM infiltration at relapse

We investigated whether there were ABL1 kinase mutations in CSF and BM samples from two of the 13 patients with CNS relapse. The first patient was a 42-year-old woman who was diagnosed with p190 BCR-ABL1-positive ALL without CNS involvement at diagnosis. After treatment with the LAL-PH-2008 protocol and with an HLA-identical allogeneic transplant, the patient achieved complete molecular remission. Eight years later, she presented with isolated leukemic CNS infiltration. A CSF sample showed p190 BCR-ABL1 ALL and the known p.L387M kinase domain mutation (imatinib-resistant but dasatinib-sensitive). The CSF blasts cleared after IT triple therapy and rescue chemotherapy with the TKI switched to dasatinib [10]. After 2 months of dasatinib treatment, this therapy was suspended due to gastrointestinal toxicity. The patient is currently in a second CR and shows good tolerance of nilotinib (400 mg twice a day). Ultra-deep NGS of two BM samples, one at diagnosis and one at CNS relapse, did not show VUS or pathogenic variants, which was consistent with the SS results.

**Fig. 3:**
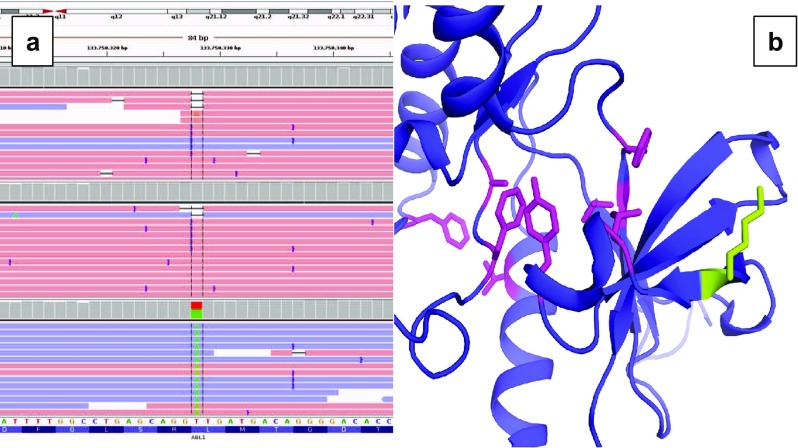
Molecular analysis. **a** Results of the Integrative Genomics Viewer analysis of the BCR-ABL1 kinase domain (exon 7) c.1159T>A (p.L387M) mutation that was found in cerebrospinal fluid blast cells at relapse (bottom). Non-mutated alleles were observed in the bone marrow sample at diagnosis (*top*) and in the bone marrow at relapse (*middle*). **b** A structural model shows the kinase domain of ABL1 (*purple*) bound to imatinib (PDB: 1IEP). The side chains of the imatinib binding residues Leu^248^, Tyr^253^, Val^289^, Thr^315^, Phe^317^, Phe^359^, Ala^380^, and Phe^382^ are shown in *pink*, and the variant amino acid Lys^245^ is *green* (Color figure online)

The second patient was a 68-year-old man diagnosed with p190 BCR-ABL1-positive ALL after a routine hematological test. Morphologic assessment and flow cytometry found no CNS involvement at diagnosis. He was treated with the LAL-OPH-2007 protocol and achieved complete morphologic and molecular response. Two years later, he experienced a combined BM and CNS relapse. The p.L387M mutation was detected in the CSF sample but, surprisingly, not in the BM sample. A second BM and CSF CR were attained with TIT, systemic chemotherapy, and dasatinib. Currently, he receives dasatinib (70 mg twice a day) without adverse effects. Ultra-deep NGS of two BM samples (one at diagnosis and one at relapse) and a CSF sample taken at relapse confirmed the pathogenic variant c.1159T>A, p.L387M only in CSF blasts in the relapse sample (Fig. 3a, bottom). No pathogenic variants were detected by NGS at low allelic frequency. In addition, we found the VUS c.733A>G, p.K245E only in the BM sample taken at diagnosis.

We used several pathogenicity predictor servers to predict the effect that the p.K245E mutation would have on the stability of the protein. Given the primary sequence of the protein, MutationTaster predicted the variant to be disease causing (score = 0.999) [11], while Polyphen-2 classified the variant as benign (score = 0.022) [12]. The Site Directed Mutation server [13] was used to create a structural model of the kinase domain from the ABL1 wild-type structure fragment from Met^225^ to Gln^498^ (PDB ID: 1IEP) in a complex with imatinib [14] with the p.K245E mutation, and it predicted the variant to be neutral and non-disease-associated based on its structure.

## Discussion

The frequency of CNS involvement in BCR-ABL1-positive ALL is variable among study populations. Notably, it is somewhat age-dependent, and its frequency is higher than in other B cell precursor ALL diseases. In pediatric and adolescent populations, CNS recurrence occurs in 3–11% of cases [15–18]. These two series of middle-aged (<55 years) and older (>55 years) adults showed CNS relapse frequencies of 7 and 14%, respectively, which is in agreement with the findings of other groups [19–21] . No single clinical variable was predictive of CNS relapse in our study, and there was a trend for prolonged OS in patients with CNS relapse vs. those with relapses at other sites.

We found the pathogenic variant p.L387M in the CSF blast cells of two patients with CNS recurrence. Notably, this mutation was not detected in BM samples at diagnosis or at relapse in the second patient. We may not have detected a mutation in BCR-ABL1 in the BM because the disease level was minimal at the time of CNS relapse (as measured using multiparametric flow cytometry). It seems likely that the selective emergence of mutated clones only in the CNS is associated with CNS imatinib concentrations that are too low to effectively prevent CNS relapse. In contrast, the independent mutation observed at BM relapse in the second patient is more likely to be associated with other mechanisms of resistance to imatinib. The mechanism(s) of resistance in CSF relapse patients that involve BCR-ABL1 mutations could be either imatinib-dependent or imatinib-independent, since the imatinib concentrations in CSF are lower than in plasma.

The mutations identified at relapse were sensitive to dasatinib and nilotinib. These TKIs, particularly dasatinib, show better penetration into the CSF than other TKIs. Based on these results, it seems prudent to perform an NGS mutational study of the BCR-ABL1 kinase domain in CSF blast cells, despite the difficulties of extracting RNA from leukocytes in the CSF. This should be integrated into the work-up protocol of these patients in order to select the best TKI based on clonal evolution of the CSF blast cells. This technique allows the identification of newly emergent clones in the CNS with high sensitivity (∼s1% mutated); notably, such mutations would be undetectable by routine SS.

In silico tools such as MutationTaster, PolyPhen-2, and Site Directed Mutator helped us to investigate the pathogenicity of the VUS p.K245E. The consensus of the in silico results plus the external position of the lysine (i.e., its location away from the imatinib binding site; Fig. 3b) suggests to us that the variant is most likely benign. Further functional studies could provide new insights into the pathogenicity of the VUS.

In summary, in BCR-ABL1 ALL patients treated with imatinib and chemotherapy, CNS relapse was an important feature despite CNS prophylaxis. Taken together, the results suggest that genomic analysis of the BCR-ABL1 kinase domain in cells from CSF should be integrated into the study of these patients in order to detect independent mutations that could be exclusive to this site.
